# Case Report: Parsonage-Turner syndrome due to SEPTIN9 mutation: report of an Italian family with childhood onset and review of the literature

**DOI:** 10.3389/fped.2025.1589397

**Published:** 2025-08-07

**Authors:** Luca Bosisio, Matteo Cataldi, Marina Grandis, Barbara Tappino, Monica Traverso, Francesco Germano, Lino Nobili, Chiara Fiorillo

**Affiliations:** ^1^ Department of Neuroscience, Rehabilitation, Ophthalmology, Genetics, Maternal and Child Health, Department of Neuroscience (DINOGMI), University of Genoa, Genoa, Italy; ^2^ Child Neuropsychiatry Unit, IRCCS Istituto Giannina Gaslini, Genoa, Italy; ^3^ Neurology Clinic, IRCCS, Ospedale Policlinico San Martino, Genova, Italy; ^4^ Central Laboratory of Analysis, IRCCS Istituto Giannina Gaslini, Genoa, Italy; ^5^ UOC Medical Genetic, IRCCS Istituto Giannina Gaslini, Genoa, Italy; ^6^ Unit of Neurology and Neurophisiolgy, EO Ospedali Galliera, Genoa, Italy

**Keywords:** Parsonage-Turner syndrome, *SEPTIN9*, children neuropathy, HNA, mutation

## Abstract

**Aim:**

Parsonage-Turner syndrome, also known as neuralgic amyotrophy affects the brachial plexus and includes idiopathic (INA) and rare hereditary forms (HNA). Mutations in the *SEPTIN9* gene, which encodes a cytoskeletal GTPase, have been implicated in HNA. While Parsonage-Turner syndrome is typically adult-onset, with stress often acting as a trigger, the presentation in children is less acknowledged.

**Methods:**

We report a case of 9-year-old girl with brachial plexus neuritis who carries a *SEPTIN9* missense mutation inherited from her father. We conducted a literature review to explore early-onset cases and gain insight into the disease's progression over time.

**Results:**

Patient presented with episodic intense pain and severe weakness in her right upper limb since age 5 years. Central nervous system involvement and inflammatory polyneuropathy were excluded. Neurological assessment showed weakness and muscle atrophy in the right shoulder girdle. Dysmorphic features, such as long nasal bridge, hypertelorism, and epicanthal folds, were also noted. Her father reported a similar episode in the past without investigations. *SEPTIN9* gene sequencing revealed the missense mutation (c.262C>T; p.Arg88Trp) in both individuals. The review of 109 patients with hereditary neuropathy linked to *SEPTIN9* mutations revealed a mean age of onset at 13 years, though the average time from symptom onset to diagnosis was 22 years. The syndrome typically follows a relapsing-remitting course, but monophasic and progressive forms are also described.

**Conclusion:**

Clinicians should consider HNA in children with asymmetric upper limb weakness and dysmorphic features, especially with a family history of upper limb neuralgia. Early diagnosis can improve long-term outcomes and avoid unnecessary tests.

## Introduction

Neuralgic amyotrophy (NA) is a rare neurological disease affecting the brachial plexus, first identified in 1948 by Parsonage and Turner (1).

The disorder may present monophasic, relapsing-remitting or, less frequently, slowly progressive course. Subjects typically experience an abrupt onset of intense pain in the upper limbs, predominantly in scapulohumeral and arm regions, which is then followed, after days to weeks, by the development of muscle weakness and, in some cases, muscle atrophy. Attacks are usually unilateral but may also occur bilaterally (2, 3). Two variants of NA have been described: the idiopathic neuralgic amyotrophy (INA) and the hereditary neuralgic amyotrophy (HNA), an autosomal dominant disorder (4, 5). From the genetic point of view, several missense mutations and pathogenic duplications of *SEPTIN9* gene have been reported (2, 6). Here we describe the clinical, electrophysiological, and genetic data of a pediatric HNA patient and provide a literature review of neuropathy phenotypes associated with *SEPTIN9* mutation.

## Methods

### Case report

Clinical data collection, neurological examination, and nerve conduction studies were conducted at Gaslini Children Hospital by pediatric neurologists (LB, MC and CF). Genetic studies were conducted by geneticists (BT, MT) by Sanger sequencing of the coding regions of the *SEPTIN9* gene.

### Search strategy

We performed a comprehensive search of PubMed/MEDLINE. The search terms were a) “*SEPTIN9*”, and B) “*SEPT9*”.

### Inclusion and exclusion criteria

We selected studies published in peer-reviewed journals. We included papers with at least one patient carrying a *SEPTIN9*variant and presenting with a peripheral nervous system (PNS) disorder. Only original case reports and case series were considered, as they provide individual-level clinical details. In contrast, review articles and meta-analyses were excluded to avoid duplication and ensure consistency in the data extraction process. Studies that were not available online or from which critical clinical or genetic data could not be extracted were also excluded.

### Study selection

Two reviewers (LB and MC) analyzed 652 manuscripts by title and abstracts from January 1993 to June 2023. Although SEPTIN9 mutations were first recognized as causative for hereditary neuralgic amyotrophy in 2005, we extended the search period back to January 1993 to identify earlier reports of clinically relevant cases that may have been genetically characterized in later studies or retrospectively associated with SEPTIN9. A total of 41 studies potentially eligible for inclusion were selected. After full-text evaluation, 15 manuscripts were included (2–16). Eligibility was assessed according to inclusion and exclusion criteria. Any discrepancies between the reviewers’ selection were resolved by a third reviewer (CF) consensus. If multiple publications shared the same pedigree, we evaluated only the latest paper or the one we considered most accurate for the purposes of our review. HNA symptomatic cases not genetically tested but with a family history of *SEPTIN9* gene mutation were equally considered variant carriers.

### Data extraction and synthesis

We extracted the following clinical features for each patient: sex, diagnosis, disease family history, disease onset and relapse age, diagnosis age, clinical features, lower limbs involvement, dysmorphic features, neurophysiological examination, disease course, residual neurological signs or symptoms and genetic variant.

## Results

### Case presentation

**Figure 1:**
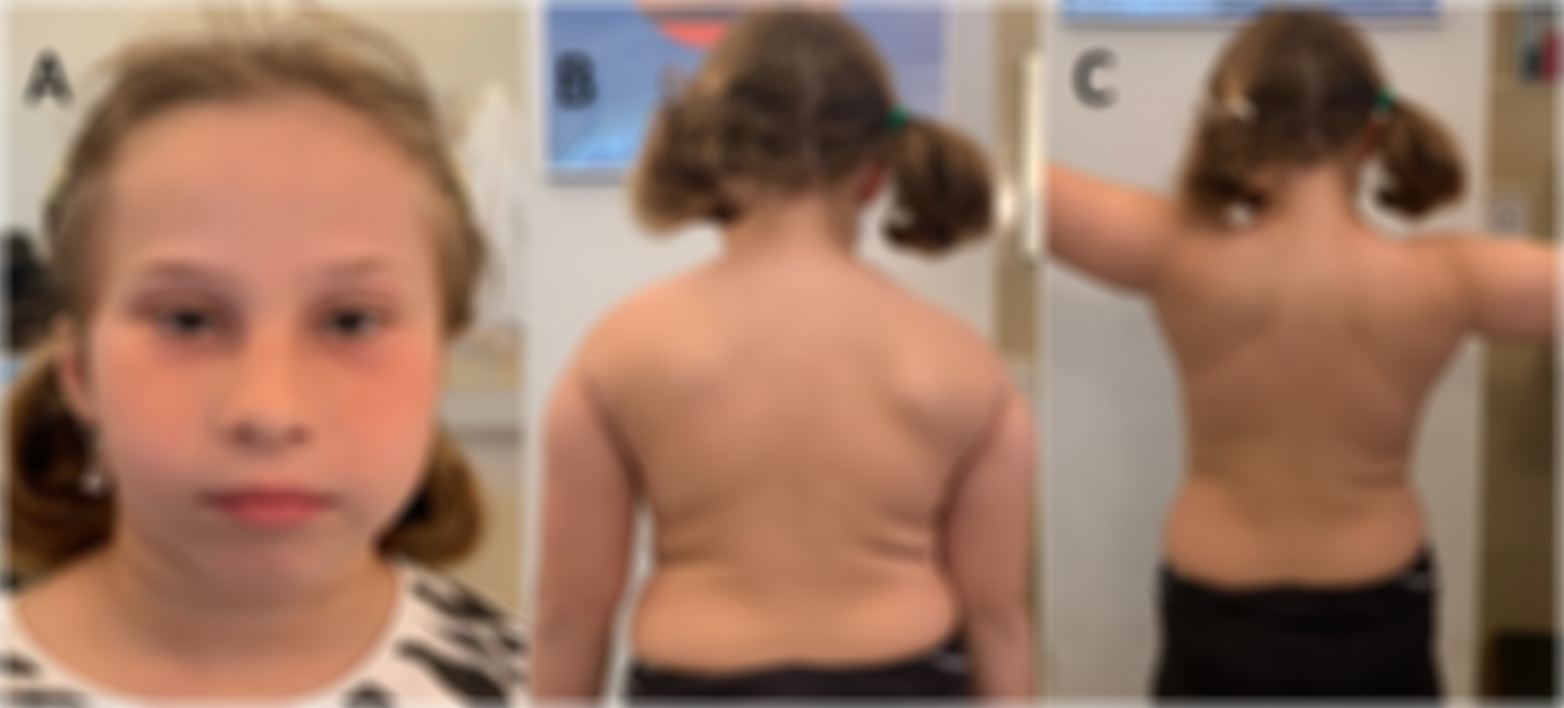
Clinical features of the patient at age 9 years. **(A)** The minor dysmorphic features including hypotelorism, shortened palpebral fissures, epicanthal fold, long nasal bridge, and neck skin folds; **(B,C)** the neuromuscular findings including bilateral winged scapula, right sloped shoulder and lesser strength in elevation of the right arm.

A 9-year-old Italian girl with normal development and good health came to our institute the age of 5 years for 2 previous episodes of pain and weakness on the right shoulder girdle and arm occurred in the past months. These episodes were described, without any physical or infectious triggers, lasting several weeks and with spontaneous remission of the pain but with residual limitation of the right arm elevation. Neurological examination showed mild bilateral atrophy, and hypotonia of the scapular girdle, predominant in the right side. Muscle strength in right deltoids and levator scapula muscles was reduced (Medical Research Council scale for muscle strength, MRC, 4) with difficulties in elevation of the right arm (Figures 1B,C). Minor dysmorphic features were noted including long nasal bridge, small oral openings, hypertelorism, epicanthal folds, and neck skin folds (Figure 1A). Cranial nerves were spared. Muscle strength was normal in lower limbs as well as sensation and coordination. Cognitive functions, vision and hearing were normal. Pain or paresthesia were not present during the evaluation. Nerve conduction studies (NCS), performed several months after the last pain episode, showed normal motor conduction velocities and Compound Muscle Action Potential (CMAP) amplitudes in the median, ulnar, tibial, and peroneal nerves bilaterally, as well as normal sensory conduction velocities in the median, ulnar, and sural nerves on both sides. Giving the normal NCS study and neglecting the unspecific pain symptom reported in such a small age patient, we suspected an inherited myopathy, but genetic tests (CGH-array and NGS genetic panel for congenital myopathy) were negative.

During the follow up visits, hypotonia and atrophy of the scapular girdle persisted, and we also detected atrophy of both pectoral muscles (more evident on the right side), hyporeflexia of the biceps and triceps reflexes and mild hyperlordosis. No sensory disturbances or pain were observed. A repeat nerve conduction study was performed at the age of 8, confirming normal conduction velocities and amplitudes of both sensory and motor potentials in the upper and lower limbs. However, an asymmetry of the F response from the ulnar nerves was detected, with a lower persistence of the F waves in the affected side. Electromyography (EMG) revealed signs of chronic denervation of right deltoid muscle including presence of polyphasic MUP with increased duration.

A closer look at the family history revealed that also the child's father suffered from two episodes of upper limb pain and weakness in the past: the first occurred at age 10 years and it was clinically characterized by muscle weakness in his right upper limb. At the age of 36 years, he had a relapse apparently triggered by a mild compression applied to his left arm during sleep. Paresthesias, pain and weakness in the left hand were detected. The patient clinically improved over months but a mild atrophy of the left thenar eminence remained. In suspicion of an early onset of a HNA, we performed a Sanger sequencing for *SEPTIN9* gene, which showed a heterozygous pathogenic variant (c.262C>T; p.Arg88Trp) (RefSeq: NM_006640.5 in GenBank) in both child and father.

### Review of HNA patients with SEPTIN9 mutations

We found 32 pedigrees (109 patients) with mutations in the *SEPTIN9* gene and a HNA clinical neurological phenotype, with the only exception of one family reported as Charcot-Marie-Tooth (CMT) (8). The mean age at disease onset, extrapolated by available retrospective information, was 13 years while the mean age at syndrome diagnosis was 35 years. In only 22 cases the disease course was available: we observed a clear prevalence of relapsing and remitting disorder over the monophasic or primarily progressive form. Clinical manifestations exclusively involved the upper limbs, except for three cases: two patients who received a diagnosis of CMT and one HNA patient with lower limbs involvement. The most common symptoms in the cohort included shoulder weakness followed by upper limbs pain. Fourteen patients had residual signs, mostly shoulder or scapular girdle atrophy, during remission stages. The nerve conduction studies or EMG investigation was performed on 14 patients, data were available in Table 1. Dysmorphic features were clearly reported in 45 patients. The most common signs were hypertelorism, skin folds in the neck and arms, and short stature (Table 1; Supplementary Table S1). Therapy history was available in only 7 patients: 3 patients received treatment with intravenous immunoglobulins with benefit in reducing the attack duration (7, 13, 15). The remaining 4 patients were treated with other drugs, and one of the two patients treated with steroids showed a positive effect from the treatment (3, 9). As per genotype, the most common mutations are the recurrent missense c.262C>T (Arg88Trp) and several duplications of the *SEPTIN9* gene or parts of it (Table 1). Rarer mutations are the 6missense 278 C>T p.Ser93Phe and the private c.-131G>C in the untranslated region of the gene identified in the first HNA family (11). Lately another variant of unknown significance c.1406T>C p.Val469Ala has been described in two patients with clinical diagnosis of CMT (8). Additional details and related references are available in the Supplementary Table S1.

**Table 1:** General, clinical, genetic, neurophysiological, and dysmorphic features of the 109 SEPTIN9 patients included in our literature review.

General data		
	Mean	Range
Age disease onset (years)	13	0–40
Age disease diagnosis (years)	35	2.5–69
	Number of subjects	
Age disease onset <18 years	23/33	
Male	49	
Female	60	
HNA diagnosis	107/109	
CMT diagnosis	2/107	
Clinical Data		
Monophasic disease course	3/22	
Relapsing-remitting disease course	16/22	
Progressive disease course	3/22 (included CMT)	
Acute motor signs (upper limbs)	24/31	
Acute pain (upper limbs)	18/31	
Acute sensitive sings (upper limbs)	12/31	
vocal cords involvement	7/31	
Lower limbs involvement	3/31 (included CMT)	
Residual neurological sign	15/20	
NCS/EMG		
Axonal and demyelinating neuropathy	7/14	
Denervation	5/14	
Axonal neuropathy	1/14	
Brachial plexopathy (unclear whether diagnosed by NCS or EMG)	1/14	
Dysmorphic features		
Hypotelorism	32	
Skin folds of the neck or arms	11	
Short stature	10	
Microstomia	7	
Epicanthal folds	5	
Small and shaped ears	3	
Cleft palate	2	
Renal cysts	2	
Blepharophimosis	2	
Thin, downward sloping eyebrows	2	
Ptosis	2	
Low-positioned ears	2	
Macroglossia	1	
Finger furrows	1	
Cleft uvula	1	
Narrow face	1	
Pectus excavatum	1	
SEPTIN9 finding		
c.262C>T (p.Arg88Trp)	68/109	
Gene Duplications	29/109	
c.278 C>T (p.Ser93Phe)	7/109	
c.-131G>C	2/109	
c.1406T>C (p.Val469Ala)	2/109 (CMT)	

HNA, hereditary neuralgic amyotrophy; CMT, Charcot-Marie-Tooth; NCS, nerve conduct studies; EMG, electromyography.

## Discussion

Parsonage-Turner syndrome is typically considered a disorder of the adult age. Accordingly, in the review of Van Alfen et al. describing 44 patients with HNA, a median age onset of 28 years is reported (17). Conversely, in our revision the age at disease onset was frequently reported in childhood (23/33) including 12 patients with symptoms before 6 years of age. (Table 1; Supplementary Table S1) The average time from clinical onset to syndrome diagnosis is 22 years. This could be a risk factor toward a worse clinical outcome, especially related to an increased risk for exposure to external triggers that may facilitate a relapses such as physical exertion, emotional stress or infectious illness. According to the proposed guidelines and HNA core features (18), our patient showed the typical HNA phenotype due to the *SEPTIN9* mutation: a pediatric onset, a family history suggestive of brachial neuralgia, and the presence of typical dysmorphic features (hypertelorism and skin folds in the neck). Moreover, despite her young age and the absence of disease relapses, the child suffered from residual atrophy even after recovering from the first neuritis episode.

Dysmorphic features could be a helpful marker in suspecting HNA; in fact, they were found in the entire population, except for two patients (43/45). These include long nasal bridge, small oral openings (microstomia), hypertelorism, epicanthal folds, and neck skin folds and are easilyrecognizable even in small children. Although nerve conduction studies (NCS) and electromyography (EMG) are essential for characterizing peripheral nerve involvement in neuralgic amyotrophy, they are not always performed in pediatric patients during the initial evaluation, particularly when symptoms are mild or have already resolved. Invasive procedures such as needle EMG are often deferred in very young children unless clearly indicated. In our case, a full electrodiagnostic study including EMG was completed at follow-up, confirming chronic denervation.

This was also reflected in our review, where NCS and EMG investigations proved useful in demonstrating denervation in the upper limbs, with sparing of the lower limbs; indeed, lower limb involvement was reported in only one patient with HNA and in two cases diagnosed with Charcot-Marie-Tooth disease. Most of mutations identified in our review are missense mutations or duplication of *SEPTIN9* gene, with different size and location often with unique breakpoints (2). Notably, *SEPTIN9* is rich in Alu sequences that might have mediated the occurrence of duplications. Apparently, there is no genotype-phenotype correlation, and both missense mutation or duplications have a similar clinical outcome.

A further SEPTIN9 missense variant c.1406T>C (p.Val469Ala), has been recently described in a autosomal dominant family with a CMT1 phenotype (8) without dysmorphic features. If this shall be confirmed, it is possible that *SEPTIN9* gene is involved in a broader spectrum of neuropathies (8). How SEPTIN9 mutations cause HNA is still debated. *SEPTIN9* belongs to the family of genes coding for Septins, a group of conserved GTP-binding proteins involved in cytoarchitecture regulation. Septins combine to form oligomeric complexes that assemble into filaments; and the Septin 9 protein promotes microtubules binding (19, 20). It has been hypothesized that mutations block SEPTIN9 filaments assembly in the cytoskeleton and this pathomechanism is a common base in the molecular etiology of other neuropathies (21).

This study has some limitations. Although we conducted a comprehensive literature review, it is possible that some relevant articles were missed, particularly those published in non-indexed journals or not available in English. Furthermore, our search was limited to studies published up to June 2023, and additional cases or relevant findings may have been reported since then. Future updates of the literature may help refine the phenotypic spectrum and natural history of HNA associated with SEPTIN9 mutations.

## Conclusion

Despite the rarity of Parsonage Turner brachial plexopathy in children, the typical clinical features combined with family history and the presence of dysmorphic features should lead the clinician to search for a *SEPTIN9* gene mutation.

## Data Availability

The original contributions presented in the study are included in the article/Supplementary Material, further inquiries can be directed to the corresponding author.
