# Supplementary material for: Case Report: Parsonage-Turner syndrome due to SEPTIN9 mutation: report of an Italian family with childhood onset and review of the literature
Source: Front Pediatr. 2025 Aug 7;13:1589397. doi: 10.3389/fped.2025.1589397 (PMC12367472; doi:10.3389/fped.2025.1589397)
Supplement: Supplementary file 1 [file Table1.docx]

| **General Data** | | | |
| --- | --- | --- | --- |
|  | *Mean* | | *Range* |
| Age disease onset (years) | 13 | | 0 - 40 |
| Age disease diagnosis (years) | 35 | | 2.5 - 69 |
|  | *Number of subjects* | | |
| Age disease onset < 18 years | 23/33 | | |
| Male | 49 | | |
| Female | 60 | | |
| HNA diagnosis | 107/109 | | |
| CMT diagnosis | 2/107 | | |
| **Clinical Data** | | | |
| Monophasic disease course | 3/22 | | |
| Relapsing-remitting disease course | 16/22 | | |
| Progressive disease course | 3/22 (included CMT) | | |
| Acute motor signs (upper limbs) | 24/31 | | |
| Acute pain (upper limbs) | 18/31 | | |
| Acute sensitive sings (upper limbs) | 12/31 | | |
| vocal cords involvement | 7/31 | | |
| Lower limbs involvement | 3/31 (included CMT) | | |
| Residual neurological sign | 15/20 | | |
| **NCS/EMG** | | | |
| Axonal and demyelinating neuropathy | 7/14 | | |
| Denervation | 5/14 | | |
| Axonal neuropathy | 1/14 | | |
| Brachial plexopathy (unclear whether diagnosed by NCS or EMG) | 1/14 | | |
| **Dysmorphic Features** | | | |
| Hypotelorism | | 32 | |
| Skin folds of the neck or arms | | 11 | |
| Short stature | | 10 | |
| Microstomia | | 7 | |
| Epicanthal folds | | 5 | |
| Small and shaped ears | | 3 | |
| Cleft palate | | 2 | |
| Renal cysts | | 2 | |
| Blepharophimosis | | 2 | |
| Thin, downward sloping eyebrows | | 2 | |
| Ptosis | | 2 | |
| Low-positioned ears | | 2 | |
| Macroglossia | | 1 | |
| Finger furrows | | 1 | |
| Cleft uvula | | 1 | |
| Narrow face | | 1 | |
| Pectus excavatum | | 1 | |
| **SEPTIN9 finding** | | | |
| c.262C>T (p.Arg88Trp) | | 68/109 | |
| Gene Duplications | | 29/109 | |
| c.278 C>T (p.Ser93Phe) | | 7/109 | |
| c.-131G>C | | 2/109 | |
| c.1406T>C (p.Val469Ala) | | 2/109 (CMT) | |
